# An Optimization-Based Approach to Social Network Group Decision Making with an Application to Earthquake Shelter-Site Selection

**DOI:** 10.3390/ijerph16152740

**Published:** 2019-07-31

**Authors:** Hengjie Zhang, Fang Wang, Huali Tang, Yucheng Dong

**Affiliations:** 1 Business School, Hohai University, Nanjing 211100, China; 2 Business School, State Key Laboratory of Hydraulics and Mountain River Engineering, Sichuan University, Chengdu 610065, China

**Keywords:** social network, group decision making, additive preference relations, consistency, earthquake shelter-site selection

## Abstract

The social network has emerged as an essential component in group decision making (GDM) problems. Thus, this paper investigates the social network GDM (SNGDM) problem and assumes that decision makers offer their preferences utilizing additive preference relations (also called fuzzy preference relations). An optimization-based approach is devised to generate the weights of decision makers by combining two reliable resources: in-degree centrality indexes and consistency indexes. Based on the obtained weights of decision makers, the individual additive preference relations are aggregated into a collective additive preference relation. Further, the alternatives are ranked from best to worst according to the obtained collective additive preference relation. Moreover, earthquakes have occurred frequently around the world in recent years, causing great loss of life and property. Earthquake shelters offer safety, security, climate protection, and resistance to disease and ill health and are thus vital for disaster-affected people. Selection of a suitable site for locating shelters from potential alternatives is of critical importance, which can be seen as a GDM problem. When selecting a suitable earthquake shelter-site, the social trust relationships among disaster management experts should not be ignored. To this end, the proposed SNGDM model is applied to evaluate and select earthquake shelter-sites to show its effectiveness. In summary, this paper constructs a novel GDM framework by taking the social trust relationship into account, which can provide a scientific basis for public emergency management in the major disasters field.

## 1. Introduction

There are many group decision making (GDM) problems in practice, and a large number of GDM approaches have been reported in the literature [1,2,3,4,5,6,7,8,9]. For example, Altuzarra et al. [1] devised a consensus building framework for analytic hierarchy process (AHP) from a Bayesian perspective. Fei et al. [10] proposed a new multi-criteria decision making method based on Dempster–Shafer evidence theory and Vlse Kriterijuska Optimizacija I Komoromisno Resenje (VIKOR). Han et al. [11] reported an interval-valued Pythagorean prioritized operator-based game theoretical framework and applied it in the multicriteria GDM. Wu et al. [12] proposed the concept of flexible linguistic expressions and applied it to the linguistic GDM problem. Zhang et al. [13] proposed a linguistic distribution-based optimization approach for large-scale GDM with comparative linguistic information. Zhang et al. [14] applied the GDM model to failure mode and effect analysis.

It should be noted that the social network analysis (SNA) has appeared as a key technique in modern sociology in recent years [15,16,17]. The SNA has been adopted in GDM to model the trust relationships among a group of decision makers. For instance, Wu et al. [18] put forward a visual interaction consensus model for social network GDM (SNGDM) with trust propagation. Pérez et al. [19] investigated SNGDM with linguistic trustworthiness-based induced ordered weighted averaging (OWA) operators. In these SNGDM works, the social network is the only source to generate weights of decision makers. It may be unreasonable because the weights of decision makers depend on multiple factors. To deal with this issue, Wu and Chiclana [20] developed a consensus model for SNGDM, where the weights of decision makers are produced by a linear combination of two reliable resources: the trust degrees in the social network and the consensus level among decision makers. However, the linear combination coefficient is not discussed in their work. In addition, Dong et al. [21] proposed a consensus strategy adding a minimum number of interactions in the social network to form a consensus based on leadership. Ureña et al. [16] brought forward a new similarity-based influence social network that leverages the knowledge of the crowds to model the public opinion dynamic and to reach consensus among the different agents involved in the decision making process. Zhang et al. [17] developed a consensus framework based on SNA to deal with non-cooperative behaviors. Recently, Dong et al. [15] provided a comprehensive literature review regarding SNGDM.

This paper investigates the SNGDM problem, which aims to help decision makers choose the best alternative or rank alternatives from best to worst. To solve the SNGDM problem, many approaches have been reported (see the previous paragraph). In the SNGDM problem, the weights of decision makers play an important role. However, we find that the social network is the only source to generate weights of decision makers in most works associated with SNGDM. In the actual SNGDM problem, the weights of decision makers often depend on multiple aspects. To our knowledge, there is only one SNGDM model focused on combination weights [20], which linearly combines two reliable resources: the trust degrees and the consensus level. The setting of the linear combination coefficient is the core problem in SNGDM. However, the linear combination coefficient is not discussed in the existing literature, and it is directly given.

Motivated by the challenges to fill the above research gap and inspired by the works associated with SNGDM [16,20], this paper proposes a novel SNGDM framework and assumes that decision makers express their preferences using additive preference relations (also fuzzy preference relations). In the novel SNGDM framework, the weights of decision makers are determined based on two reliable resources: in-degree centrality indexes derived from the social network and the consistency indexes of preference relations. In particular, an optimization-based model is devised to obtain the weighting coefficient of in-degree centrality indexes and consistency indexes, which seeks to maximize the consensus level among the decision makers. Based on the obtained weighting coefficient, the comprehensive weights of decision makers are generated from the in-degree centrality indexes and the consistency indexes. Then, the individual additive preference relations are aggregated into a collective additive preference relation according to the obtained weights of decision makers. Further, the alternatives are ranked from best to worst based on the collective additive preference relation.

This paper constructs a novel SNGDM framework by considering the social trust relationship among decision makers, which is of great significance in helping disaster management experts effectively evaluate and select emergency shelter-sites and constructs a scientific basis for public health emergency management in the major disasters field. In the following, we discuss the practical and societal implications of the proposal by applying it in the earthquake shelter-site selection problem.

In recent years, many major natural hazard-caused disasters have occurred worldwide, which caused huge losses of property and personnel. Earthquakes are among the most terrifying and destructive of all natural hazard-caused disasters, which can arguably lead to incalculable environmental damage, construction damage, loss of life, and population displacement [22]. China and Japan are the most earthquake-prone countries in the world, and have experienced significant damage events in recent years, such as the 2008 Wenchuan earthquake (China), the 2010 Yushu earthquake (China), the 2011 earthquake of the Pacific coast of Tōhoku (Japan), and the 2013 YaAn earthquake (China). Among them, the 2008 Wenchuan earthquake and the 2011 earthquake of the Pacific coast of Tōhoku caused the greatest loss of life. In the following, we list some of the largest earthquakes in the world in recent years (see Table 1).

Earthquakes destroy or damage houses and infrastructure, which calls for arrangement of temporary establishments to provide immediate evacuation and shelter to disaster-affected people [23]. Shelters provide safety, security, climate protection, and resistance to disease and ill health and are thus vital for disaster-affected people. On one hand, shelters facilitate the provision of food, accommodation, and medical care to affected people. On the other hand, shelters protect the population from possible dangers arising at later phases of recovery. It is critically important for locating shelters from potential alternatives to select a suitable site, as selection of a suitable site affects the performance of an evacuation plan. Many works for earthquake shelter-site evaluation and selection have been reported. For example, Trivedi [24] presented a multi-criteria decision making model to evaluate determinants of shelter site selection based on decision making trial and evaluation laboratory (DEMATEL). Xu et al. [25] proposed a two-stage consensus reaching model for large-scale multi-attribute GDM and applied it to earthquake shelter selection. Song et al. [26] devised an approach that combines the strength of the qualitative flexible multiple criteria (QUALIFLEX) method in sustainable shelter-site selection under uncertainty. In [27], a mixed integer linear program was formulated to address the shelter area selection problem. The above analysis shows that many approaches have been reported to select emergency shelters. Among them, group decision theory and method is an effective method to evaluate and locate emergency shelters. In the emergency shelter-site evaluation and selection problem, identifying disaster management experts is a very important issue. The experts can be selected according to their educational background, level of professional skills, length of service, and participation in emergency management. Moreover, social trust relationships exist widely among disaster management experts, which have an important impact on disaster management. On one hand, social trust relationships should be an important source of the weights of disaster management experts. For disaster management, experts who received high trust values from others should be assigned high weights. On the other hand, during the discussion process of disaster management, an expert will refer to the opinions of the experts he/she trust. However, existing approaches fail to address the social trust relationships among disaster management experts. Therefore, the proposed SNGDM approach is adopted to assess and select the emergency shelter-site to verify its application value.

We arrange the remainder of this paper as follows. Section 2 introduces the basic knowledge. Section 3 presents the SNGDM problem and proposes its resolution framework. Section 4 provides a case study regarding earthquake shelter-site selection to show the validity of the proposed SNGDM framework, and a comparison analysis is also offered in this section. Finally, the concluding remarks are presented in Section 5.

## 2. Preliminaries

This section provides preliminary information regarding the social network analysis and additive preference relations.

### 2.1. Social Network Analysis

SNA (social network analysis) has emerged as a key technique in modern sociology, and it focuses on the relationships between social entities such as families, corporations, or nations [28]. The SNA based methodology is very useful to model social trust relationships among a group of decision makers, which has been adopted widely in GDM [17,29].

A social network contains three main elements: the set of actors, the relations themselves, and the actor attributes, which are described in Table 2.

The trust relationships among decision makers (denoted as $E=\{e_{1},e_{2},\ldots ,e_{m}\}$) are represented by a sociometric, which is defined as below [17,30].

**Definition** **1.** *A sociometric $S={\left(s_{ij}\right)}_{m\times m}$ on E is a relation in E×E with membership function $u_{S}$: E×E→[0, 1], and $u_{S}(e_{i},\;e_{j})=s_{ij}$, where $s_{ij}\in [0,\;1]$ denotes the trust degree that decision maker $e_{i}$ assigns to $e_{j}$.*

**Example** **1.** *The trust relationship is represented in direct graph form as in Table 2 with the following sociometric $S={\left(s_{ij}\right)}_{5\times 5}$.*

$$S=\left(\begin{array}{ccccc} 0 & 0.7 & 0.8 & 0 & 0.9\\ 0 & 0 & 0.55 & 0 & 0.65\\ 0 & 0 & 0 & 0.75 & 0\\ 0.8 & 0 & 0.9 & 0 & 0\\ 0 & 0.85 & 0 & 0.8 & 0 \end{array}\right)$$

### 2.2. Additive Preference Relations

Many formats of preference relations have been reported, such as multiplicative preference relations, the additive preference relations (also called fuzzy preference relations), and linguistic preference relations. The different preference relations can be transformed into each other using transformation functions [3]. Without loss of generality, we use additive preference relations to denote the opinions of individuals, which are introduced below [31,32].

**Definition** **2.** *The matrix $F={\left(f_{ij}\right)}_{n\times n}$, where $f_{ij}\in [0,1]$ denotes the preference degree of alternative $x_{i}$ over $x_{j}$, and $f_{ij}+f_{ji}=1$ for i,j=1,2,…,n, is called an additive preference relation.*

**Example** **2.** *The additive preference relation over alternatives $\left\{x_{1},x_{2},x_{3},x_{4},x_{5}\right\}$ can be represented as below.*

$$F^{\mathrm{example}\;2}=\left(\begin{array}{ccccc} 0.5 & 0.7 & 0.8 & 0.6 & 0.85\\ 0.3 & 0.5 & 0.78 & 0.65 & 0.7\\ 0.2 & 0.22 & 0.5 & 0.88 & 0.6\\ 0.4 & 0.35 & 0.12 & 0.5 & 0.55\\ 0.15 & 0.3 & 0.4 & 0.45 & 0.5 \end{array}\right)$$

In $F^{\mathrm{example}\;2}$, $f_{12}^{\mathrm{example}\;2}=0.7$ means that the preference degree of the alternative $x_{1}$ over $x_{2}$ is 0.7, and $f_{45}^{\mathrm{example}\;2}=0.55$ denotes that the preference degree of the alternative $x_{4}$ over $x_{5}$ is 0.55. Similarity, the rest of the elements in $F^{\mathrm{example}\;2}$ can be explained in this way.

The concept of consistency of additive preference relations is presented as follows [33].

**Definition** **3.** *Let $F={\left(f_{ij}\right)}_{n\times n}$ be as before, the consistency index (CI) based on additive transitivity for numerical preference relations, $F={\left(f_{ij}\right)}_{n\times n}$, is defined as:*

(1) $$CI(F)=1-\frac{2}{3n(n-1)(n-2)}\sum_{i=j=k=1,i\neq j\neq k}^{n}\left|f_{ij}+f_{jk}-f_{ik}-0.5\right|$$

Clearly, CI(F) varies from 0 to 1. A bigger CI(F) value indicates a higher consistency of $F={\left(f_{ij}\right)}_{n\times n}$. In practice, it is difficult to offer a full consistency additive preference relation (i.e., CI(F)=1) for a decision maker. To deal with this issue, a consistency threshold (denoted as δ∈[0,1]) is defined to judge whether the consistency level of additive preference relations is acceptable. If CI(F)≥δ, then the consistency level of $F={\left(f_{ij}\right)}_{n\times n}$ is acceptable; otherwise it is not acceptable.

## 3. Decision Problem and Its Resolution Framework

In this section, we formally present the SNGDM problem and design a framework to solve it.

### 3.1. The Presentation of the SNGDM Problem

As mentioned in Section 1, the social network among decision makers should be an essential element in the GDM problem. In addition, it is difficult for decision makers to provide their opinions over alternatives using an accurate way owing to the complexity and uncertainty of the real-world GDM problem. Meanwhile, additive preference relation is an effective way to convey the uncertainty information of decision makers, which is utilized in this paper to represent the opinions of decision makers.

Herein, the SNGDM problem is formally proposed as follows. Let $E=\{e_{1},e_{2},\ldots ,e_{m}\}$ (m≥2) be a finite set of decision makers and $\lambda ={\left(\lambda_{1},\lambda_{2},\ldots ,\lambda_{m}\right)}^{T}$ be the associated weight vector over decision makers E, where $\lambda_{k}\geq 0$ (k=1,2,…,m) denotes the weight of decision maker $e_{k}$ and $\sum_{k=1}^{m}\lambda_{k}=1$. Let $S={\left(s_{ij}\right)}_{m\times m}$ be the sociometric among E, where $s_{ij}\in [0,1]$ denotes the trust degree of decision maker $e_{i}$ assigns to $e_{j}$. Let $X=\{x_{1},x_{2},\ldots ,x_{n}\}$ (n≥2) be a finite set of alternatives. In this paper, we assume that decision makers $e_{k}$ adopt additive preference relations to express their preferences over alternatives X, $F^{k}={\left(f_{ij}^{k}\right)}_{n\times n}$ (k=1,2,…,m), where $f_{ij}^{k}\in [0,1]$ is the preference degree of alternative $x_{i}$ over $x_{j}$ provided by decision maker $e_{k}$.

The decision problem is how to assist decision makers to obtain a collective solution in the SNGDM with additive preference relations.

### 3.2. Resolution Framework for SNGDM Problems

The weights of decision makers play an important role in GDM/SNGDM problems.

**Figure 1:**
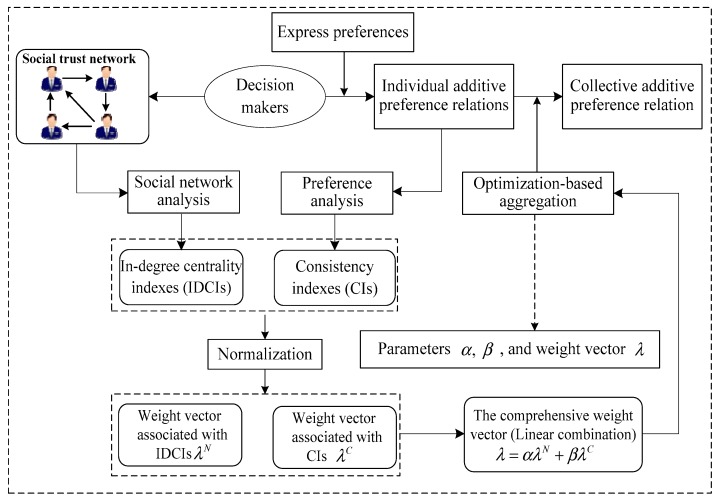
The optimization-based social network group decision making (SNGDM) framework.

There are many methods to determine the weights of decision makers [34,35]. Notably, Wu and Chiclana [20] determined the weights of decision makers by linearly combining two reliable resources: trust degree and consensus level in the SNGDM. Although this method was very useful for determining the weights of decision makers, the linear combination coefficient was not discussed. Inspired by the work of [20], we propose an optimization-based approach to the SNGDM problem, which is presented in Figure 1.

In the optimization-based SNGDM framework, in-degree centrality indexes of decision makers are generated using SNA from a social trust network. Meanwhile, the consistency indexes of decision makers are produced using the preference analysis method. Then, an optimization-based model is designed to determine the weights of decision makers by combining the above two reliable resources.

(1) Reliable resources of weights

Here, we introduce two reliable resources of weights: in-degree centrality indexes and consensus indexes.

(a) The weights derived from in-degree centrality indexes.

Let $S={\left(s_{ij}\right)}_{m\times m}$ be the above, then the in-degree centrality index, $IDCI^{k}$, associated with decision maker $e_{k}$ ∈ *E*, can be computed as below [34]:

(2) $$IDCI^{k}=\frac{1}{m-1}\sum_{i=1,\;i\neq k}^{m}s_{ik}.$$

In general, a larger value of the in-degree centrality index indicates a larger weight of the decision maker. According to this principle, we present the following way to obtain the weights of decision makers.

Let $\lambda^{N}={\left(\lambda_{1}^{N},\;\lambda_{2}^{N},\;\ldots ,\;\lambda_{m}^{N}\right)}^{T}$ be the weights of decision makers derived from in-degree centrality indexes, where

(3) $$\lambda_{k}^{N}=\frac{IDCI^{k}}{\sum_{i=1}^{m}IDCI^{i}}.$$

Clearly, $\lambda_{k}^{N}\in [0,1]$ and $\sum_{k=1}^{m}\lambda_{k}^{N}=1$.

(b) The weights derived from consistency indexes.

Let $CI(F^{k})$ be the consistency index of the additive preference relation $F^{k}={\left(f_{ij}^{k}\right)}_{n\times n}$ obtained using Equation (1). A larger consistency index of the additive preference relation implies that the more logical the decision information of the decision maker is. So, the decision maker with a large consistency index should assign a high weight. Based on this basic idea, we present the following approach to obtain the weights of decision makers.

Let $\lambda^{C}={\left(\lambda_{1}^{C},\;\lambda_{2}^{C},\;\ldots ,\;\lambda_{m}^{C}\right)}^{T}$ be the weight vector of decision makers derived from consistency indexes, where

(4) $$\lambda_{k}^{C}=\frac{CI(F^{k})}{\sum_{i=1}^{m}CI(F^{i})}.$$

Clearly, $\lambda_{k}^{C}\in [0,1]$ and $\sum_{k=1}^{m}\lambda_{k}^{C}=1$.

(2) Determine the weights of decision makers

In the above, we analyzed two reliable resources of weights of decision makers. The core issue in the SNGDM is how to combine these two reliable resources so as to obtain the comprehensive weights of decision makers. Let $\lambda ={\left(\lambda_{1},\ldots ,\lambda_{m}\right)}^{T}$ be the comprehensive weight vector of decision makers, then $\lambda_{k}$ can be obtained by:

(5) $$\lambda_{k}\;=\;\alpha \lambda_{k}^{N}\;+\;\beta \lambda_{k}^{C}$$

where α,β are the linear combination coefficients, and α+β=1 (α,β≥0).

In Equation (5), $\lambda_{k}\in [0,1]$ and $\sum_{k=1}^{m}\lambda_{k}=1$.

Before presenting the optimization-based model to determine α and β, we introduce some relative concepts.

Let $F^{c}={\left(f_{ij}^{c}\right)}_{n\times n}$ be the collective additive preference relation derived from $\left\{F^{1}={\left(f_{ij}^{1}\right)}_{n\times n},\ldots ,F^{m}={\left(f_{ij}^{m}\right)}_{n\times n}\right\}$, where

(6) $$\left\{ \begin{array}{l} f_{ij}^{c}=\sum_{k=1}^{m}\lambda_{k}\times f_{ij}^{k}=\sum_{k=1}^{m}(\alpha \lambda_{k}^{N}+\beta \lambda_{k}^{C})\times f_{ij}^{k},\;i\leq j\\ f_{ij}^{c}=1-f_{ji}^{c},\;i>j \end{array}\right..$$

The consensus level is used to indicate the current level of consensus in consensus reaching process, and several different consensus measure approaches have been suggested [36]. The use of consensus measure approaches will not influence the proposed SNGDM framework. Without loss of generality, we compute the consensus level by computing the similarity degree between the individual and collective preferences.

The consensus level of decision maker $e_{k}$, $CL(e_{k})$, is computed by:

(7) $$CL(e_{k})=1-d(F^{k},\;F^{c})$$

where

(8) $$d(F^{k},\;F^{c})=\frac{1}{n(n-1)}\sum_{i=1}^{n}\sum_{j=1,j\neq i}^{n}\left|f_{ij}^{k}-f_{ij}^{c}\right|.$$

The consensus level among $\left\{e_{1},\;e_{2},\;\ldots ,\;e_{m}\right\}$, $CL\{e_{1},\;e_{2},\;\ldots ,\;e_{m}\}$ is defined by:

(9) $$\begin{array}{l} CL\{e_{1},\;e_{2},\;\ldots ,\;e_{m}\}=\frac{1}{m}\sum_{k=1}^{m}CL(e_{k})\\ =1-\frac{1}{mn(n-1)}\sum_{k=1}^{m}\sum_{i=1}^{n}\sum_{j=1,j\neq i}^{n}\left|f_{ij}^{k}-f_{ij}^{c}\right| \end{array}$$

If $CL\{e_{1},e_{2},\ldots ,e_{m}\}=1$, then all decision makers are at a full consensus with the collective preference. Otherwise, a higher $CL\{e_{1},e_{2},\ldots ,e_{m}\}$ value indicates a higher consensus level among all the decision makers.

Note 1: The proposed optimization-based SNGDM framework is a general framework, and the use of the consensus measure method will not change any essence of the framework. Without loss of generality, in this paper, the consensus level among all decision makers is calculated by averaging the consensus levels of all decision makers. It may be interesting in the future to use the consensus measure method that integrates the weights of decision makers in the proposed optimization-based SNGDM framework.

In GDM, achieving a consensus is very important [37]. A high consensus level indicates the highly accepted group solution to the GDM is generated. Naturally, we hope the consensus level among all decision makers is as good as possible, that is,

(10) $$\max(1-\frac{1}{m}\sum_{k=1}^{m}d(F^{k},\;F^{c})).$$

Meanwhile, the known weight information on α and β can be typically constructed using the following basic forms:

(i) Weak ranking: $\Omega_{1}=\{\alpha \geq \beta \;\mathrm{or}\;\beta \geq \alpha \}$
(ii) Strict ranking: $\Omega_{2}=\{\alpha -\beta \geq \gamma \;\mathrm{or}\;\beta -\alpha \geq \gamma \}$ (γ≥0)
(iii) Ranking with multiples: $\Omega_{3}=\{\alpha \geq \gamma \times \beta \;\mathrm{or}\;\beta \geq \gamma \times \alpha \}$ (γ≥0)

Without loss of generality, we use Ω to denote the set of known information about parameters α and β provided by decision makers. In particular, $\Omega =\Omega_{1}\cup \Omega_{2}\cup \Omega_{3}$.

Based on the above analysis, an optimization-based model can be constructed:

(11) $$\begin{array}{l} \max CL\{e_{1},\;e_{2},\;\ldots ,\;e_{m}\}=1-\frac{1}{m}\sum_{k=1}^{m}d(F^{k},\;F^{c})\\ s.t.\left\{ \begin{array}{l} f_{ij}^{c}=\sum_{k=1}^{m}\lambda_{k}\times f_{ij}^{k},\;i,j=1,2,\ldots ,n\\ f_{ij}^{c}+f_{ji}^{c}=1,i,j=1,2,\ldots ,n\\ \lambda_{k}=\alpha \lambda_{k}^{N}+\beta \lambda_{k}^{C},\;k=1,2,\ldots ,m\;\\ \alpha +\beta =1\\ 0\leq \alpha ,\beta \leq 1,\alpha ,\beta \in \Omega \; \end{array}\right. \end{array}$$

In Equation (11), $\lambda ={\left(\lambda_{1},\ldots ,\lambda_{m}\right)}^{T}$ and $F^{c}={\left(f_{ij}^{c}\right)}_{n\times n}$ are decision variables. Solving Equation (11) produces the optimal solution to $\lambda ={\left(\lambda_{1},\ldots ,\lambda_{m}\right)}^{T}$ and $F^{c}={\left(f_{ij}^{c}\right)}_{n\times n}$. Obviously, Equation (11) is a nonlinear programming. For the convenience of solving, transforming Equation (11) into a linear programming model is necessary. In the following, we convert Equation (11) into a linear programming model, which is described as Theorem 1.

**Theorem** **1.** *Equation (11) can be equivalently transformed into the following linear programming model:*

(12) $$\begin{array}{l} \max\left\{1-\frac{1}{mn(n-1)}\sum_{k=1}^{m}\sum_{i=1}^{n}\sum_{j=1,j\neq i}^{n}a_{ij}^{k}\right\}\\ s.t.\left\{ \begin{array}{l} f_{ij}^{k}-f_{ij}^{c}\leq a_{ij}^{k},\;i,j=1,\ldots ,n;\;j\neq i\;\;\;(a)\\ -f_{ij}^{k}+f_{ij}^{c}\leq a_{ij}^{k},\;i,j=1,\;,n;\;j\neq i\;\;\;\;(b)\\ f_{ij}^{c}=\sum_{k=1}^{m}\lambda_{k}\times f_{ij}^{k},\;i,j=1,\ldots ,n\;\;\;\;\;\;\;\;(c)\\ f_{ij}^{c}+f_{ij}^{c}=1,\;i,j=1,\ldots ,n\;\;\;\;\;(d)\\ \lambda_{k}=\alpha \lambda_{k}^{N}+\beta \lambda_{k}^{C},\;k=1,\ldots ,m\;\;\;(e)\\ \alpha +\beta =1\;\;\;(f)\\ 0\leq \alpha ,\;\beta ,\;a_{ij}^{k}\leq 1,\;k=1,\ldots ,m;\;i,j=1,\ldots ,n;\alpha ,\beta \in \Omega \;\;\;(g) \end{array}\right. \end{array}.$$

**Proof** **.** In Equation (12), constraints (a) and (b) guarantee that $|f_{ij}^{k}-f_{ij}^{c}|\leq a_{ij}^{k}$. According to the objective function of Equation (12), any feasible solution with $|f_{ij}^{k}-f_{ij}^{c}|<a_{ij}^{k}$ is not the optimal solution to Equation (12). Thus, constraints (a) and (b) guarantee $a_{ij}^{k}=|f_{ij}^{k}-f_{ij}^{c}|$. Therefore, Equation (11) can be equivalently transformed into Equation (12). This completes the proof of Theorem 1.

(3) Obtain the ranking of alternatives

Let $P={\left(p_{1},\;p_{2},\;\ldots ,\;p_{n}\right)}^{T}$ be the preference vector derived from $F^{c}={\left(f_{ij}^{c}\right)}_{n\times n}$, where the preference value over alternative $x_{i}$ is computed by:

(13) $$p_{i}=\frac{2}{n(n-1)}\sum_{j=1,\;j\neq i}^{n}f_{ij}^{c}.$$

The larger value of $p_{i}$, the better the alternative $x_{i}$ is. So, the alternatives $\left\{x_{1},\;x_{2},\;\ldots ,\;x_{n}\right\}$ can be ranked from best to worst based on $P={\left(p_{1},\;p_{2},\;\ldots ,\;p_{n}\right)}^{T}$.

The detailed procedures of the proposed SNGDM framework are described as the following algorithm.


**Algorithm I:**
**Input:** Decision makers, $E=\{e_{1},e_{2},\ldots ,e_{m}\}$; sociometric among E, $S={\left(s_{ij}\right)}_{m\times m}$; a set of alternatives, $X=\{x_{1},x_{2},\ldots ,x_{n}\}$; additive preference relations, $F^{k}={\left(f_{ij}^{k}\right)}_{n\times n}$(k=1,2,…,m).**Output:** Weight vector over decision makers E, $\lambda ={\left(\lambda_{1},\lambda_{2},\ldots ,\lambda_{m}\right)}^{T}$; the ranking over X.**Step 1:** Using Equation (2) obtains in-degree centrality indexes, i.e., $IDCI^{k}=\frac{1}{m-1}\sum_{i=1,\;i\neq k}^{m}s_{ik}$
(k=1,2,…,m). Further, we have that $\lambda_{k}^{N}=\frac{IDCI^{k}}{\sum_{i=1}^{m}IDCI^{i}}$ according to Equation (3).**Step 2:** Use Equation (1) to obtain the consistency levels, i.e., $CI(F)=1-\frac{2}{3n(n-1)(n-2)}\sum_{i=j=k=1,i\neq j\neq k}^{n}\left|f_{ij}+f_{jk}-f_{ik}-0.5\right|$. Then, according to Equation (4), we have that $\lambda_{k}^{C}=\frac{CI(F^{k})}{\sum_{i=1}^{m}CI(F^{i})}$.**Step 3:** According to Equation (5), we have $\lambda_{k}=\alpha \lambda_{k}^{N}+\beta \lambda_{k}^{C}$. The collective preference relation is obtained using Equation (6), i.e., $f_{ij}^{c}=\sum_{k=1}^{m}\lambda_{k}\times f_{ij}^{k}=\sum_{k=1}^{m}(\alpha \lambda_{k}^{N}+\beta \lambda_{k}^{C})\times f_{ij}^{k}$ for i≤j; and $f_{ij}^{c}=1-f_{ji}^{c}$ for i>j. Using Equation (9) generates the collective consensus level among decision makers, i.e., $CL\{e_{1},\;e_{2},\;\ldots ,\;e_{m}\}=\frac{1}{m}\sum_{k=1}^{m}CL(e_{k})=1-\frac{1}{mn(n-1)}\sum_{k=1}^{m}\sum_{i=1}^{n}\sum_{j=1,j\neq i}^{n}\left|f_{ij}^{k}-f_{ij}^{c}\right|$.**Step 4:** Based on step 3, model (11) is constructed. Using theorem 1, model (11) is converted into a linear programming model, i.e., model (12). Solving model (12) obtains $\lambda^{*}={\left(\lambda_{1}^{*},\lambda_{2}^{*},\ldots ,\lambda_{m}^{*}\right)}^{T}$ and $F^{c,*}={\left(f_{ij}^{c,*}\right)}_{n\times n}$.**Step 5:** Use Equation (13) to obtain the collective preference vector over alternatives $P={\left(p_{1},\;p_{2},\;\ldots ,\;p_{n}\right)}^{T}$, where $p_{i}=\frac{2}{n(n-1)}\sum_{j=1,\;j\neq i}^{n}f_{ij}^{c,*}$. According to P, the ranking of alternatives X is generated. Output the ranking of alternatives.

## 4. Case study and Comparative Analysis

In this section, a case study and a comparative analysis are offered to verify the effectiveness of the proposed SNGDM model.

### 4.1. Case Study

Here, a case study regarding earthquake shelter-site selection problem is provided to show the application value of the proposal.

A 6.0-magnitude earthquake shook Changning County of Yibin City, Sichuan Province, China on June 17, 2019. The epicenter, with a depth of 16 km, was monitored at 28.34 degrees north latitude and 104.90 degrees east longitude, according to the China Earthquake Networks Center (CENC) [38]. The quake affected eight counties, including Changning County, Gong County, and Gao County. The quake was felt strongly in the major districts of neighboring Chongqing Municipality, which was not too far from the epicenter, and a few residential houses were damaged, although no casualties were reported, according to the Chongqing Municipal Emergency Management Bureau. The quake was also felt by Chengdu City and a few counties of Zhaotong City in Yunnan Province.

As of 16:00 on 20 June 2019, 13 people had died and 220 were injured. Among the injured, 153 people were receiving treatment in hospitals, and around 243,880 people were affected [39]. As of 16:00 on 20 June, 2019, the quake had severely damaged 46,000 houses and slightly damaged more than 110,000 houses. As of 13:00 on 23 June, there were more than 44 aftershocks of magnitude 3.0 or above, including three earthquakes of magnitude 5.0 to 5.9, four earthquakes of magnitude 4.0 to 4.9, and 37 earthquakes of magnitude 3.0 to 3.9.

To relieve the suffering of the disaster-affected population, emergency shelter construction is of critical importance. Suppose that a committee including four disaster management experts (denoted as $e_{1}$, $e_{2}$, $e_{3}$, and $e_{4}$) was formed to be responsible for the emergency shelter construction in Changning County. The first step is to select shelter-site. After preliminary screening, four sites are chosen for further evaluation, which are denoted as $x_{1}$, $x_{2}$, $x_{3}$, and $x_{4}$. The disaster management experts $e_{k}$ (k=1,2,…,4) express their preferences on the four alternative shelter-sites using additive preference relations $F^{k}$.

$$F^{1}=\left(\begin{array}{cccc} 0.5 & 0.2 & 0.6 & 0.4\\ 0.8 & 0.5 & 0.9 & 0.7\\ 0.4 & 0.1 & 0.5 & 0.3\\ 0.6 & 0.3 & 0.7 & 0.5 \end{array}\right)F^{2}=\left(\begin{array}{cccc} 0.5 & 0.7 & 0.9 & 0.5\\ 0.3 & 0.5 & 0.6 & 0.7\\ 0.1 & 0.4 & 0.5 & 0.8\\ 0.5 & 0.3 & 0.2 & 0.5 \end{array}\right)$$

$$F^{3}=\left(\begin{array}{cccc} 0.5 & 0.3 & 0.5 & 0.7\\ 0.7 & 0.5 & 0.1 & 0.3\\ 0.5 & 0.9 & 0.5 & 0.25\\ 0.3 & 0.7 & 0.75 & 0.5 \end{array}\right)F^{4}=\left(\begin{array}{cccc} 0.5 & 0.25 & 0.15 & 0.65\\ 0.75 & 0.5 & 0.6 & 0.8\\ 0.85 & 0.4 & 0.5 & 0.5\\ 0.35 & 0.2 & 0.5 & 0.5 \end{array}\right)$$

Meanwhile, the social network among the four disaster management experts is as follows:


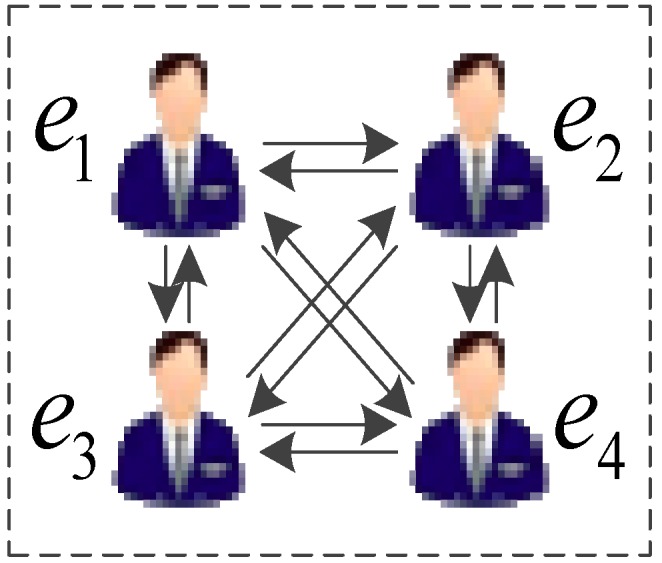


The sociometric, S, associated with the above social network is provided below:

$$S=\left(\begin{array}{rrrr} - & 0.65 & 0.52 & 0.44\\ 0.5 & - & 0.3 & 0.6\\ 0.52 & 0.61 & - & 0.9\\ 0.3 & 0.45 & 0.8 & - \end{array}\right).$$

Then, use Equation (2) to obtain the in-degree centrality index, $IDCI^{k}$, associated with disaster management experts $e_{k}$ (k=1,2,3,4):

$$IDCI^{1}=\frac{1}{3}(0.5+0.52+0.3)=0.44,$$

$$IDCI^{2}=\frac{1}{3}(0.65+0.61+0.45)=0.57,$$

$$IDCI^{3}=\frac{1}{3}(0.52+0.3+0.8)=0.54,$$

$$IDCI^{4}=\frac{1}{3}(0.44+0.6+0.9)=0.6467.$$

Use Equation (3) to obtain the weights of disaster management experts derived from in-degree centrality indexes:

$$\lambda_{1}^{N}=\frac{0.44}{0.44+0.57+0.54+0.6467}=\frac{0.44}{2.1967}=0.2,$$

$$\lambda_{2}^{N}=\frac{0.57}{0.44+0.57+0.54+0.6467}=\frac{0.57}{2.1967}=0.2595,$$

$$\lambda_{3}^{N}=\frac{0.54}{0.44+0.57+0.54+0.6467}=\frac{0.54}{2.1967}=0.246,$$

$$\lambda_{4}^{N}=\frac{0.6467}{0.44+0.57+0.54+0.6467}=\frac{0.6467}{2.1967}=0.2945,$$

i.e., $\lambda^{N}={\left(0.2,\;0.2595,\;0.246,\;0.2945\right)}^{T}$.

Apply Equations (1) and (4) to produce the weights of disaster management experts derived from consistency indexes:

$$\lambda^{C}={\left(0.3077,0.2359,0.2,0.2564\right)}^{T}.$$

In this example, we set α−β≥0.3. Based on the optimization-based model (12), we have that:

$$\max\left\{1-\frac{1}{48}\sum_{k=1}^{4}\sum_{i=1}^{4}\sum_{j=1,j\neq i}^{4}a_{ij}^{k}\right\}$$

s.t.

$$0.2-f_{12}^{c}\leq a_{12}^{1},\;0.6-f_{13}^{c}\leq a_{13}^{1},\;0.4-f_{14}^{c}\leq a_{14}^{1},\;0.9-f_{23}^{c}\leq a_{23}^{1},\;0.7-f_{24}^{c}\leq a_{24}^{1},\;0.3-f_{34}^{c}\leq a_{34}^{1},$$

$$0.7-f_{12}^{c}\leq a_{12}^{2},\;0.9-f_{13}^{c}\leq a_{13}^{2},\;0.5-f_{14}^{c}\leq a_{14}^{2},\;0.6-f_{23}^{c}\leq a_{23}^{2},\;0.7-f_{24}^{c}\leq a_{24}^{2},\;0.8-f_{34}^{c}\leq a_{34}^{2},$$

$$0.3-f_{12}^{c}\leq a_{12}^{3},\;0.5-f_{13}^{c}\leq a_{13}^{3},\;0.7-f_{14}^{c}\leq a_{14}^{3},\;0.1-f_{23}^{c}\leq a_{23}^{3},\;0.3-f_{24}^{c}\leq a_{24}^{3},\;0.25-f_{34}^{c}\leq a_{34}^{3},$$

$$0.25-f_{12}^{c}\leq a_{12}^{4},\;0.15-f_{13}^{c}\leq a_{13}^{4},\;0.65-f_{14}^{c}\leq a_{14}^{4},\;0.6-f_{23}^{c}\leq a_{23}^{4},\;0.8-f_{24}^{c}\leq a_{24}^{4},\;0.5-f_{34}^{c}\leq a_{34}^{4}.$$

$$-0.2+f_{12}^{c}\leq a_{12}^{1},\;-0.6+f_{13}^{c}\leq a_{13}^{1},\;-0.4+f_{14}^{c}\leq a_{14}^{1},\;-0.9+f_{23}^{c}\leq a_{23}^{1},\;-0.7+f_{24}^{c}\leq a_{24}^{1},\;-0.3+f_{34}^{c}\leq a_{34}^{1},$$

$$-0.7+f_{12}^{c}\leq a_{12}^{2},\;-0.9+f_{13}^{c}\leq a_{13}^{2},\;-0.5+f_{14}^{c}\leq a_{14}^{2},\;-0.6+f_{23}^{c}\leq a_{23}^{2},\;-0.7+f_{24}^{c}\leq a_{24}^{2},\;-0.8+f_{34}^{c}\leq a_{34}^{2},$$

$$-0.3+f_{12}^{c}\leq a_{12}^{3},\;-0.5+f_{13}^{c}\leq a_{13}^{3},\;-0.7+f_{14}^{c}\leq a_{14}^{3},\;-0.1+f_{23}^{c}\leq a_{23}^{3},\;-0.3+f_{24}^{c}\leq a_{24}^{3},\;-0.25+f_{34}^{c}\leq a_{34}^{3},$$

$$-0.25+f_{12}^{c}\leq a_{12}^{4},\;-0.15+f_{13}^{c}\leq a_{13}^{4},\;-0.65+f_{14}^{c}\leq a_{14}^{4},\;-0.6+f_{23}^{c}\leq a_{23}^{4},\;-0.8+f_{24}^{c}\leq a_{24}^{4},\;-0.5+f_{34}^{c}\leq a_{34}^{4}.$$

$$f_{12}^{c}=0.2\cdot \lambda_{1}+0.7\cdot \lambda_{2}+0.3\cdot \lambda_{3}+0.25\cdot \lambda_{4},\;f_{13}^{c}=0.6\cdot \lambda_{1}+0.9\cdot \lambda_{2}+0.5\cdot \lambda_{3}+0.15\cdot \lambda_{4},$$

$$f_{14}^{c}=0.4\cdot \lambda_{1}+0.5\cdot \lambda_{2}+0.7\cdot \lambda_{3}+0.65\cdot \lambda_{4},\;f_{23}^{c}=0.9\cdot \lambda_{1}+0.6\cdot \lambda_{2}+0.1\cdot \lambda_{3}+0.3\cdot \lambda_{4},$$

$$f_{24}^{c}=0.7\cdot \lambda_{1}+0.7\cdot \lambda_{2}+0.3\cdot \lambda_{3}+0.8\cdot \lambda_{4},\;f_{34}^{c}=0.3\cdot \lambda_{1}+0.8\cdot \lambda_{2}+0.25\cdot \lambda_{3}+0.5\cdot \lambda_{4},$$

$$f_{12}^{c}+f_{21}^{c}=1,\;f_{13}^{c}+f_{31}^{c}=1,\;f_{14}^{c}+f_{41}^{c}=1,\;f_{23}^{c}+f_{32}^{c}=1,\;f_{24}^{c}+f_{42}^{c}=1,\;f_{34}^{c}+f_{43}^{c}=1,$$

$$\lambda_{1}=0.2\cdot \alpha +0.3077\cdot \beta ,\;\lambda_{2}=0.2595\cdot \alpha +0.2359\cdot \beta ,$$

$$\lambda_{3}=0.246\cdot \alpha +0.2\cdot \beta ,\;\lambda_{4}=0.2945\cdot \alpha +0.2564\cdot \beta ,\;\alpha +\beta =1\;,\;\alpha -\beta \geq 0.3,$$

$$0\leq \alpha ,\;\beta ,\;a_{ij}^{k}\leq 1,\;k=1,\ldots ,4;\;i,j=1,\ldots ,4.$$

Solving the above model, we have that

α=0.65 and β=0.35.

Further, the comprehensive weights of disaster management experts are generated:

$$\lambda ={\left(0.2377,\;0.2512,\;0.2299,\;0.2812\right)}^{T}.$$

Meanwhile, the collective additive preference relation is obtained, that is,

$$F^{c}=\left(\begin{array}{cccc} 0.5 & 0.3627 & 0.5258 & 0.5644\\ 0.6373 & 0.5 & 0.5564 & 0.6362\\ 0.4742 & 0.4436 & 0.5 & 0.4703\\ 0.4356 & 0.3638 & 0.5297 & 0.5 \end{array}\right).$$

Using Equation (13) ($p_{i}=\frac{2}{n(n-1)}\sum_{j=1,\;j\neq i}^{n}f_{ij}^{c}$) obtains $p_{i}$ (i=1,2,3,4) from $F^{c}$:

$$p_{1}=\frac{2}{n(n-1)}(f_{12}^{c}+f_{13}^{c}+f_{14}^{c})=\frac{1}{6}(0.3627+0.5258+0.5644)=0.4843,$$

$$p_{2}=\frac{2}{n(n-1)}(f_{21}^{c}+f_{23}^{c}+f_{24}^{c})=\frac{1}{6}(0.6373+0.5546+0.6362)=0.61,$$

$$p_{3}=\frac{2}{n(n-1)}(f_{31}^{c}+f_{32}^{c}+f_{34}^{c})=\frac{1}{6}(0.4742+0.4436+0.4703)=0.4627,$$

$$p_{4}=\frac{2}{n(n-1)}(f_{41}^{c}+f_{42}^{c}+f_{43}^{c})=\frac{1}{6}(0.4356+0.3638+0.5297)=0.443,$$

i.e., $P={\left(0.2422,\;0.3050,\;0.2314,\;0.2215\right)}^{T}$. So, the four alternatives can be ranked from best to worst, that is, $x_{2}≻x_{1}≻x_{3}≻x_{4}$.

### 4.2. Comparative Analysis

In existing works on SNGDM, Wu and Chiclana [20] determined the weights of decision makers by linearly combining two reliable resources: trust degree and consensus level in the SNGDM. However, the linear combination coefficient was not discussed in [20], and it is assumed to be fixed.

In this section, we compared our proposal with [20] based on the data used in Section 4.1. In particular, the following cases are considered: (1) α=0.65 and β=0.35; (2) α=0.7 and β=0.3; (3) α=0.75 and β=0.25; (4) α=0.8 and β=0.2; (5) α=0.85 and β=0.15; and (6) α=0.9 and β=0.1. The comparison results are provided in Table 3. In Table 3, the weight vector over the four experts $\lambda ={\left(\lambda_{1},\ldots ,\lambda_{m}\right)}^{T}$ is obtained using Algorithm I. The collective preference relation $F^{c}={\left(f_{ij}^{c}\right)}_{n\times n}$ is generated based on Equation (6), where $f_{ij}^{c}=\sum_{k=1}^{m}\lambda_{k}\times f_{ij}^{k}$. The preference vector $P={\left(p_{1},\ldots ,p_{n}\right)}^{T}$ is yielded according to Equation (13), where $p_{i}=\frac{2}{n(n-1)}\sum_{j=1,\;j\neq i}^{n}f_{ij}^{c}$. Moreover, the consensus level is measure using Equation (9), $CL\{e_{1},\;e_{2},\;\ldots ,\;e_{m}\}=\frac{1}{m}\sum_{k=1}^{m}CL(e_{k})\;=1-$ $\frac{1}{mn(n-1)}\sum_{k=1}^{m}\sum_{i=1}^{n}\sum_{j=1,j\neq i}^{n}\left|f_{ij}^{k}-f_{ij}^{c}\right|$.

From Section 4.1, we have that α=0.65 and β=0.35. So, the result obtained under case 1 is equal to that of our proposal. From Table 3, we have that setting different values of α and β will result in different comprehensive weights of decision makers, collective additive preference relations, preference vectors, and consensus levels. Meanwhile, the consensus level obtained from our proposal (i.e., Case 1) is better than those obtained from the baseline approach (i.e., Cases 2–6).

## 5. Discussion

In this section, we compare our proposal with some existing works to show the new aspects of the proposal.

(1) Comparison with [6,40,41]. In recent years, many works on GDM have been reported. Liu et al. [6] proposed a GDM approach to deal with the preference information with multiple self-confidence levels. Liu et al. [40] reported an approach to manage consensus and self-confidence in multiplicative preference relations in GDM. Xiao et al. [41] devised a model to address personalized individual semantics and consensus in linguistic distribution GDM. Compared with these works, this paper constructs a novel GDM by taking social trust relationships into account.
(2) Comparison with [16,17,18,20]. The literature on SNGDM has grown rapidly in recent years. Zhang et al. [17] investigated non-cooperative behaviors in consensus-based multiple attribute SNGDM. Wu et al. [18] proposed a visual interaction consensus model for SNGDM with trust propagation. Ureña et al. [16] reported a social network based approach for consensus achievement in multi-person decision making. Compared with these works, the weights of decision makers are determined according to two sources (social trust network and preference relations). Wu and Chiclana [20] assigned the weights of decision makers by linearly combining trust degree and consensus level. However, the linear combination coefficient was not discussed in [20], and it is assumed to be fixed. Compared with Wu and Chiclana [20], this paper designed an optimization-based model to obtain the combination coefficient.
(3) Comparison with [24,25,42]. The GDM approaches have been widely used to solve the problem of earthquake shelter-site selection. For example, Xu et al. [25] proposed a two-stage consensus method for earthquake shelter-site selection problem. Trivedi and Singh [42] prioritized emergency shelter areas using hybrid multi-criteria decision approach. Trivedi [24] presented a multi-criteria decision approach based on DEMATEL to assess determinants of shelter site selection in disaster response. Compared these GDM based works for shelter-site selection, this paper designed a novel GDM approach by considering social trust relationships among disaster management experts for shelter-site evaluation and selection.

## 6. Conclusions

This paper investigates the SNGDM problem and develops an optimization-based SNGDM framework to solve it. The main contributions are below:

(1) The social trust relationships among decision makers have recently received attention in GDM due to their influence on decision process and results. This paper investigates the SNGDM problem and designs a novel framework to solve it.
(2) In the proposed SNGDM framework, an optimization-based approach is presented to produce the weights of decision makers from two reliable resources: the in-degree centrality indexes and the consistency indexes.

This paper provides a scientific basis for public emergency management in the major natural hazard-caused disasters. To show the effectiveness and validity of the proposed SNGDM framework, a case study regarding earthquake shelter-site selection is provided.

Meanwhile, there are two limitations of the proposal:

(1) The consensus is an essential issue in the GDM [43,44,45,46], and this issue is not studied in depth in the proposed SNGDM framework.
(2) The large-scale GDM have recently attracted the attention by researchers [13], and the decision problem is investigated under the small-scale GDM context.

In future research, we plan to design an optimization-based consensus model to deal with the large-scale SNGDM problem.

## Figures and Tables

**Table 1:** Major earthquakes in the last decade.

Date	Earthquakes	Magnitude
12 May 2008	WenChuan earthquake (China)	8.0
14 April 2010	YuShu earthquake (China)	7.1
11 March 2011	The 2011 earthquake of the Pacific coast of Tōhoku (Japan)	8.8
20 April 2013	YaAn earthquake (China)	7.0
25 April 2015	Gorkha earthquake (Nepal)	8.1
8 August 2017	JiuZhaiGou earthquake (China)	7.0
8 September 2017	The 2017 Chiapas earthquake (Mexico)	8.2
18 June 2018	The 2018 Northern Osaka Earthquake (Japan)	6.1
17 June 2019	YiBin earthquake (China)	6.0

**Table 2:** Different representation schemes in social network analysis (SNA).

Graph	Sociometric	Algebraic
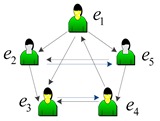	$S=\left(\begin{array}{ccccc} 0 & 1 & 1 & 0 & 1\\ 0 & 0 & 1 & 0 & 1\\ 0 & 0 & 0 & 1 & 0\\ 1 & 0 & 1 & 0 & 0\\ 0 & 1 & 0 & 1 & 0 \end{array}\right)$	$e_{1}\;R\;e_{2}$, $e_{1}\;R\;e_{3}$, $e_{1}\;R\;e_{5}$$e_{2}\;R\;e_{3}$, $e_{2}\;R\;e_{5}$, $e_{3}\;R\;e_{4}$, $e_{4}\;R\;e_{1}$, $e_{4}\;R\;e_{3}$, $e_{5}\;R\;e_{2}$, $e_{5}\;R\;e_{4}$

**Table 3:** Comparison results.

Cases	Weight Vector over Decision Maker	Collective Additive Preference Relation	Preference Vector	Consensus Level
Case 1	$\begin{array}{l} (0.2377,\;0.2512,\\ 0.2299,\;0.2812{)}^{T} \end{array}$	$\left(\begin{array}{cccc} 0.5 & 0.3627 & 0.5258 & 0.5644\\ 0.6373 & 0.5 & 0.5564 & 0.6362\\ 0.4742 & 0.4436 & 0.5 & 0.4703\\ 0.4356 & 0.3638 & 0.5297 & 0.5 \end{array}\right)$	$\begin{array}{l} (0.2422,\;0.3050,\\ 0.2314,\;0.2215{)}^{T} \end{array}$	0.8233
Case 2	$\begin{array}{l} (0.2323,\;0.2524,\\ 0.2322,\;0.2831{)}^{T} \end{array}$	$\left(\begin{array}{cccc} 0.5 & 0.3636 & 0.5251 & 0.5657\\ 0.6364 & 0.5 & 0.5536 & 0.6354\\ 0.4749 & 0.4464 & 0.5 & 0.4712\\ 0.4343 & 0.3646 & 0.5288 & 0.5 \end{array}\right)$	$\begin{array}{l} (0.2424,\;0.3042,\\ 0.2312,\;0.2213{)}^{T} \end{array}$	0.8223
Case 3	$\begin{array}{l} (0.2270,\;0.2536,\\ 0.2345,\;0.2849{)}^{T} \end{array}$	$\left(\begin{array}{cccc} 0.5 & 0.3645 & 0.5244 & 0.5670\\ 0.6355 & 0.5 & 0.5508 & 0.6347\\ 0.4756 & 0.4492 & 0.5 & 0.4721\\ 0.4330 & 0.3653 & 0.5279 & 0.5 \end{array}\right)$	$\begin{array}{l} (0.2427,\;0.3035,\\ 0.2328,\;0.2210{)}^{T} \end{array}$	0.8226
Case 4	$\begin{array}{l} (0.2215,\;0.2548,\\ 0.2368,\;0.2869{)}^{T} \end{array}$	$\left(\begin{array}{cccc} 0.5 & 0.3654 & 0.5237 & 0.5682\\ 0.6346 & 0.5 & 0.5481 & 0.6340\\ 0.4763 & 0.4519 & 0.5 & 0.4729\\ 0.4318 & 0.3660 & 0.5271 & 0.5 \end{array}\right)$	$\begin{array}{l} (0.2429,\;0.3028,\\ 0.2335,\;0.2208{)}^{T} \end{array}$	0.8222
Case 5	$\begin{array}{l} (0.2162,\;0.2560,\\ 0.2391,\;0.2887{)}^{T} \end{array}$	$\left(\begin{array}{cccc} 0.5 & 0.3663 & 0.5229 & 0.5695\\ 0.6337 & 0.5 & 0.5453 & 0.6332\\ 0.4771 & 0.4547 & 0.5 & 0.4738\\ 0.4305 & 0.3668 & 0.5262 & 0.5 \end{array}\right)$	$\begin{array}{l} (0.2431,\;0.3020,\\ 0.2343,\;0.2206{)}^{T} \end{array}$	0.8219
Case 6	$\begin{array}{l} (0.2108,\;0.2571,\\ 0.2414,\;0.2907{)}^{T} \end{array}$	$\left(\begin{array}{cccc} 0.5 & 0.3672 & 0.5222 & 0.5708\\ 0.6328 & 0.5 & 0.5425 & 0.6325\\ 0.4778 & 0.4575 & 0.5 & 0.4746\\ 0.4292 & 0.3675 & 0.5254 & 0.5 \end{array}\right)$	$\begin{array}{l} (0.2434,\;0.3013,\\ 0.2350,\;0.2204{)}^{T} \end{array}$	0.8215
